# Use of Rectus Flaps in Reconstructive Surgery for Gynecologic Cancer

**DOI:** 10.3390/curroncol31010026

**Published:** 2024-01-10

**Authors:** Oleksandra Dzyubak, Lina Salman, Allan Covens

**Affiliations:** 1 Division of Gynecologic Oncology, Department of Obstetrics & Gynecology, University of Toronto, 610 University Ave., Toronto, ON M5G 2M9, Canada; oleksandra.dzyubak@uhn.ca (O.D.); lina.salman@uhn.ca (L.S.); 2 Division of Gynecologic Oncology, Sunnybrook Health Sciences Centre, 2075 Bayview Ave., Toronto, ON M4N 3M5, Canada

**Keywords:** rectus abdominis myocutaneous flap, pelvic reconstruction, neovagina, gynecologic cancer

## Abstract

The aim of this study was to explore the outcomes of pelvic reconstruction with a rectus abdominis myocutaneous (RAM) or rectus abdominis myoperitoneal (RAMP) flap following radical surgery for gynecologic malignancy. This is a retrospective case series of all pelvic reconstructions with RAM or RAMP flap performed in a gynecologic oncology service between 1998 and 2023. Reconstructions with other flaps were excluded. A total of 28 patients were included. Most patients had vulvar cancer (*n* = 15, 53.6%) and the majority had disease recurrence (*n* = 20, 71.4%). Exenteration was the most common procedure, being carried out in 20 (71.4%) patients. Pelvic reconstruction was carried out with a RAM flap in 24 (85.7%) cases and a RAMP flap in 4 (14.3%) cases. Flap-specific complications included cellulitis (14.3%), partial breakdown (17.9%), and necrosis (17.9%). Donor site complications included surgical site infection and necrosis occurring in seven (25.0%) and three (10.7%) patients, respectively. Neovaginal reconstruction was performed in 14 patients. Out of those, two (14.3%) had neovaginal stenosis and three (21.4%) had rectovaginal fistula. In total, 50% of patients were disease-free at the time of the last follow up. In conclusion, pelvic reconstruction with RAM/RAMP flaps, at the time of radical surgery for gynecologic cancer, is an uncommon procedure. In our case series, we had a significant complication rate with the most common being infection and necrosis. The development of a team approach, with input from services including Gynecologic Oncology and Plastic Surgery should be developed to decrease post-operative complications and improve patient outcomes.

## 1. Introduction

Pelvic exenteration is a radical procedure performed in locally advanced or recurrent vulvar, cervical, or endometrial cancer and involves en-block resection of the central pelvic organs. The procedure can be classified as anterior, posterior, or total, depending on the site of the tumor [1,2,3].

In order to achieve negative margins and minimize the risk of recurrence, a large defect is created, which poses a challenge in reconstruction. High tension on the tissues, dead space prone to infection and wound break down and the loss of pelvic support can jeopardize the success of the operation and contribute to significant patient morbidity [4,5]. Anatomical distortion from radical vulvar resection can also significantly affect patient’s self-image and psychological wellbeing [6].

Weikel et al. (2017) demonstrated a correlation between the surface area of a tumor and the surgical procedure adopted, with 87% of patients undergoing pelvic exenteration having reconstruction with a myocutaneous flap [7]. A flap is defined as a unit of tissue that contains skin, subcutaneous tissue, muscle, and a vascular pedicle and is transferred from a donor site to a recipient site with its blood supply [8]. The incorporation of myocutaneous flaps into radical pelvic surgery minimizes complications, improves wound outcomes and improves patient’s quality of life [3,6,9,10].

The rectus abdominis myocutaneous (RAM) flap is widely used in gynecologic oncology and serves to provide perineal wound coverage, creation of neovagina, and obliteration of the dead space [9,11]. This flap provides large tissue bulk, reliable blood supply based on inferior epigastric artery and skin paddles which can be designed vertically, transversely, and obliquely with a wide rotational arc [9]. It decreases perineal morbidity without significantly compromising the donor site [4]. In addition, previous radiation is a common phenomenon in the treatment course of gynecologic cancers, and the RAM flap provides healthy tissue from outside of the radiation field with normal wound healing capacity [8,12]. Chessin et al. (2005) demonstrated that the use of a vertical RAM flap in patients undergoing abdominoperineal resection decreased the rate of perineal wound complications to 15.8%, from the 44% observed in the control group [13]. Complications associated with RAM flaps include wound infection, neovaginal stenosis, flap necrosis, and abdominal hernia at the donor site [5,10,14]. Alternatively, a rectus abdominis myoperitoneal (RAMP) flap, which is created from the rectus abdominis muscle, posterior sheath, and peritoneum, can be used for neovaginal reconstruction [10,15].

Utilization of myocutaneous flaps in the setting of radical resection of gynecologic malignancies leads to improvement in wound healing and quality of life references [4,6]; however, this is still an uncommon procedure with small study populations and great heterogeneity in data. In this study, we aim to report our experience with RAM and RAMP flap reconstruction performed concurrently with radical pelvic surgery at Sunnybrook Health Sciences Center.

## 2. Materials and Methods

We reviewed all cases of pelvic reconstruction with a RAM or RAMP flap following a radical surgery for gynecologic malignancy performed in the Gynecologic Oncology Department at Sunnybrook Health Sciences Centre between August 1998 and May 2023. The Sunnybrook Research Ethics Board approval was obtained. Patients were identified retrospectively through a departmental surgical registry. We included patients who underwent radical resection with reconstruction for recurrent or locally advanced vulvar, vaginal, cervical, or endometrial carcinoma. We excluded patients with ovarian cancer due to different treatment characteristics. Primary closure, skin grafts, and other types of flaps were excluded.

Patient demographics including age, body mass index (BMI), comorbidities, diagnosis, and prior exposure to radiation therapy were collected. Data pertaining to surgical characteristics included the type of resection and the type of concurrently performed procedures and reconstruction. Complications that were incurred within 30 days of surgery were identified. The emphasis was on flap-related adverse outcomes, including flap infection, partial breakdown, necrosis, neovaginal stenosis, and fistula formation, as well as donor site complications. Oncological outcomes included the rate of recurrence and status at the time of last follow up.

Clinicopathological variables were analyzed with descriptive statistics. Data on categorical variables were reported as frequencies and percentages. Continuous variables were described as median and range.

## 3. Results

### 3.1. Patient Characteristics

Between August 1998 and May 2023, 28 patients underwent pelvic reconstruction with a RAM or RAMP flap following a radical pelvic resection for gynecologic malignancy at our institution. Clinical characteristics of patients are detailed in Table 1. The median age at the time of surgery was 57 years (range 38–77). The median BMI was 27 kg/m^2^ (range 22–50.9). Diabetes was noted in two (7.1%) patients. Five (17.9%) patients were smokers at the time of surgery. Tumor diagnosis included 15 (53.6%) vulvar carcinomas, 8 (28.6%) cervical carcinomas, 2 (7.1%) endometrial carcinomas, and 3 (10.7%) vaginal carcinomas. Twenty (71.4%) patients underwent radical surgery with reconstruction for disease recurrence. Most patients (*n* = 24, 85.7%) received radiation therapy to the pelvis prior to surgery.

### 3.2. Surgical Characteristics

Surgical characteristics are listed in Table 2. In terms of the extirpative procedure, the majority of patients underwent a pelvic exenteration: eight (28.6%) had a total exenteration, eight (28.6%) had a posterior exenteration, and four (14.3%) had an anterior exenteration. Six (21.4%) patients had a radical vulvectomy only and two (7.1%) patients had a radical vaginectomy only. The median tumor size at the time of the surgery was 4.0 cm (range 0.0–10.5). Patients had one or multiple concurrent procedures, with urinary reconstruction (*n* = 14, 50%) and neovagina creation (*n* = 14, 50%) being the most common. Urinary reconstruction technique included Indiana pouch (*n* = 8), ileal conduit (*n* = 4) and Mitrofanoff procedure (*n* = 2). In the cases of posterior or total exenteration, colostomy was created in 11 (39.3%) patients. A low rectal anastomosis was done in five (17.9%) patients, all of which had a diverting loop ileostomy. The pelvic reconstructive portion of the surgery was performed by gynecologic oncology in majority of patients (*n* = 26, 92.9%), while plastic surgery performed the reconstruction in two (7.1%) patients. A RAM flap was utilized in 24 (85.7%) cases, with vertical flap (VRAM) being performed in 6 (21.4%) patients and transverse flap (TRAM) in 18 (64.3%) patients. A RAM flap was utilized in the defect closure and for neovagina creation. A RAMP flap was used for patients requiring a neovaginal reconstruction only (*n* = 4, 14.3%). The median total operative time was 480 min (range 240–780). The median estimated blood loss was 1000 mL (range 200–4500), with eight (28.6%) patients requiring an intraoperative blood transfusion. The median length of hospital admission was 16 days (range 5–39).

### 3.3. Flap Specific Complications

Recipient-site complications, involving the perineum or neovagina, and donor-site complications were captured over the whole duration of the follow up (Table 3). Minor complications included flap cellulitis (*n* = 5, 17.9%) and partial flap breakdown (*n* = 5, 17.9%), neither leading to flap loss. Five (17.9%) patients had a complete flap necrosis resulting in the loss of the flap. There were 14 patients who underwent creation of a neovagina: the RAM flap was used in 10 (71.4%) patients, and RAMP flap was used in 4 (28.6%) patients. In this population, neovaginal stenosis occurred in two (14.3%) patients. Three (21.4%) patients developed a fistula between the rectum and neovagina. A fistula developed only in patients who had a concurrent low rectal anastomosis and neovagina creation. There was no neovaginal prolapse documented in our population. Donor site complications included abdominal wall surgical site infection (*n* = 7, 25.0%) and necrosis (*n* = 3, 10.7%). There was no hernia development either at the donor or recipient site in our cohort.

Two patients died within 30 days of surgery. One patient died from septic shock, and the other died due to superior mesenteric vein thrombosis leading to extensive bowel ischemia and necrosis. Both deaths were directly related to the extirpative procedure and not to the flap reconstruction.

The median duration of follow up was 15.6 months (range 0.62–314.7). Half of the patients developed a recurrence after the radical resection with reconstruction *(n* = 14, 50%). The median time from surgery to recurrence was 9.5 months (range 1–72). Five (35.7%) recurrences were in the perineal region, three (21.4%) were in the pelvis, and six (42.9%) were at a distant site. At the time of last follow up, half of the patients were alive without evidence of disease recurrence *(n* = 14, 50%), while eight (28.6%) patients were alive with a disease recurrence. Six (21.4%) patients died, with three deaths occurring due to disease progression and three deaths arising from medical complications. Fifteen (53.6%) patients were lost to follow up.

## 4. Discussion

Pelvic exenteration has the potential to cure locally advanced or recurrent gynecological malignancies; however, this procedure carries a significant risk of morbidity, ranging between 21.3% and 94.4% [1]. The rate of complications has been shown to be lower when the extirpative procedure is combined with pelvic reconstruction with the use of a flap [12]. A systematic review and meta-analysis by Devulapalli et al. (2015) showed that primary closure was twice as likely to be associated with perineal wound complications when compared to the flap closure [4]. The benefit of myocutaneous flaps is achieved by obliteration of pelvic dead space and revascularization of previously radiated tissue [4,16]. The creation of neovagina is also associated with reduction of pelvic morbidity [17] and improved quality of life [6].

Various techniques have been used in pelvic and vaginal reconstruction following radical pelvic resection. The extent and the location of the resection guides the choice of an appropriate flap for reconstruction. Gentileschi et al. (2016) developed an algorithm to optimize pelvic reconstruction. When exenteration is performed, myocutaneous flaps such as the gracilis flap, RAM flap, or anterolateral thigh (ALT) flap are recommended to fill the dead space [18]. RAM flaps are the mainstay when it comes to pelvic reconstruction in gynecologic oncology patients. When compared to the gracilis myocutaneous flap, RAM flaps are associated with a lower rate of flap-specific and overall complications [6,14,19]. When groin or mons pubis defects are created, a deep inferior epigastric artery perforator flap (DIEP), VRAM, or ALT flap can be used. Lotus flaps and traditional V-Y flaps are best suited for the vulvoperineal area [18].

The results of our case series on pelvic reconstruction with RAM or RAMP flap confirm the feasibility of this procedure. We reviewed cases from a single institution over the span of 25 years, which allowed for a comparable population size. Devulapalli et al. (2015) reported a 35.4% pooled perineal complication rate in flap reconstruction, with the majority of analyzed flaps being RAM [4]. Common flap-specific, recipient-site complications include flap necrosis, partial flap breakdown, flap cellulitis, pelvic abscess, fistula formation, and neovaginal stenosis. Donor-site complications include infection, fascial dehiscence, and hernia formation. In the literature, the flap necrosis rate ranges between 2.2 and 18.8% [10,14,17,19,20]. In our study, 17.9% of patients experienced flap necrosis and loss, which is comparable to previous case series. RAM flaps have only one blood supply, derived from the inferior epigastric artery, and therefore, patients need to be carefully selected for this procedure. Flap loss is more likely in patients who had multiple prior abdominal incisions, have significant atherosclerotic disease, have a stoma on the same side of flap harvesting, or had traumatic flap handling during surgery. These characteristics increase the potential for damage/thrombosis to the inferior epigastric vessels [17,19]. At our center, a Doppler ultrasound of the inferior epigastric vessels is routinely performed prior to proceeding with a RAM flap reconstruction to confirm adequate vascular flow. Other complications in our population included flap infection and partial breakdown, which did not result in the loss of the flap. The median length of admission was 16 days, comparing favorably with the previously reported median of 15.6 days [4].

Half of the patients (*n* = 14) in our study underwent neovaginal reconstruction. This was achieved with the use of a RAM flap in 10 patients and a RAMP flap in 4 patients. Neovaginal stenosis occurred in two (14.3%) patients, both treated with a TRAM flap. Studies have demonstrated that in contrast to RAM flaps, RAMP flaps are more susceptible to neovaginal stenosis [10,15]. Soper et al. (2005) reported a total 13% rate of neovaginal stenosis in VRAM and TRAM flaps, in comparison to a 43% rate of stenosis with a RAMP flap [10]. Berger et al. (2012) used a VRAM flap for vaginal reconstruction and noted a 6.5% rate of stenosis [20]. The utility of a RAMP flap in neovaginal reconstruction was brought into question by the case series by Rietjens et al. (2002). Stenosis occurred in 4 out of 5 (80%) patients who underwent reconstruction with a RAMP flap, while 0 out of 5 patients treated with a RAM flap had this complication [21]. Jurado et al. (2000) performed reconstruction with a RAM flap (68.8%), gracilis myocutaneous flap (18.8%), and Singapore flap (12.5%), and their overall stenosis rate was 31.6%. There was no neovaginal stenosis in patients who had a RAMP flap reconstruction in our study. Rectovaginal fistula was reported at a rate of 6–20% in the literature [15,17]. Three (21.4%) patients in our series developed a rectovaginal fistula. This complication only occurred in patients undergoing concurrent neovaginal reconstruction and rectal anastomosis. Inflammation, vaginal and rectal surgery, and a history of radiation are all risk factors for fistula development [22], which likely explains the higher risk in our patient group.

With the use of RAM or RAMP flaps, there is also a possibility of donor site complications. Our results show that seven (25%) patients had a surgical site infection, while three (10.7%) had surgical site necrosis. Berger et al. (2012) reported a 47.8% rate of wound separation [20], while Soper et al. (2005) had a 13% rate of wound separation [17]. There were no hernias at the donor site in our study. The hernia rate in the literature is reported to be between 0 and 29% [10,17]. Additionally, there was no statistically significant difference in hernia development between primary and flap closures in the meta-analysis performed by Devulapalli et al. (2016) [4]. These findings are reassuring, as the use of the flap should not be at the expense of other complications.

Difficulties may arise when bilateral ostomies are necessary, such as in the case of a total exenteration requiring concurrent urinary and stool diversion. However, this is not an absolute contraindication to the RAM flap [9,23,24]. Skin-shift post-flap harvesting needs to be taken into consideration when marking the position of the ostomy. At our center, the ostomy site is marked before the procedure and again after the flap is harvested to modify the location if required, based upon abdominal wall mobilization. We have commonly created ileal conduits on the ipsilateral side of the flap harvest and have not experienced problems with stoma prolapse. Moller et al. (2006) described their experience with placing the urinary stoma on the same side as the donor site for the VRAM flap [25]. Urinary reconstruction was carried out as a Bricker-type ileal conduit, raised through internal oblique muscle lateral to the right rectus muscle sheath. Their outcomes were comparable to the ileal conduit raised through the rectus muscle [25].

Pelvic reconstruction at the time of radical resection may improve the quality of life, self-esteem, and sexual function of patients [6]. About 80–100% of patients report satisfaction with the aesthetics of reconstruction, while 25–85% report intercourse [6]. Soper et al. (2005) found that at the 12-month follow up, 58% of surviving patients engaged in intercourse [10]. We did not formally examine sexual function in our sample of patients with neovagina. Most studies are limited in their psychosexual assessment post-surgery for gynecologic malignancy [17].

Risk factors contributing to poor wound healing include smoking, diabetes, elevated BMI, and previous radiation therapy [1,20]. Radiation-induced fibrosis and decreased oxygen delivery create a poor wound healing environment [1]. Although the majority of patients (85.7%) in our case series had previous radiation therapy to the pelvis, flap necrosis rates were not higher than previous reports [10,14,17,19,20].

Gynecologic Oncology performed pelvic reconstruction in the majority of the patients (*n* = 26, 92.9%) in our study. This is unique, as this portion of the procedure is commonly performed by Plastic Surgery [10,13,14]. Gynecologic Oncology skilled in flap creation is in an ideal position to coordinate the resection and reconstruction portions of the operation, contributing to the comprehensive intraoperative and post operative care of the patient [17]. While our results demonstrate comparable complications to other publications, supporting Gynecologic Oncology’s role in performing pelvic reconstruction, there is significant room for improvement in morbidity, including a reduction in flap and donor site complications.

Traditionally, pelvic exenteration is performed as an open procedure and is associated with a significant risk of complications, slow recovery, and mortality [26]. With the advances in minimally invasive gynecologic surgery, technical feasibility of laparoscopic and robotic-assisted pelvic exenteration has been proven in several reports [26,27]. Early results have been encouraging, indicating less blood loss and a shorter hospital stay [26,27]. Bizzarri et al. (2019) performed laparoscopic and robotic-assisted pelvic exenteration in 23 women [28]. They report a more favorable postoperative complication rate when compared to the open procedure described in the literature. Additionally, they found less blood loss and a shorter hospital stay, at the expense of a longer operative time [28]. However, the benefit of a minimally invasive approach would be negated when a RAM flap is required for pelvic reconstruction. Harvest of the RAM flap requires a vertical incision and division of the anterior rectus sheath, which can lead to infection, seroma, and hernia formation, compromising the minimally invasive approach to exenteration [29]. Recently, robotic-assisted harvest of a RAM flap has been developed and is described by Ibrahim et al. (2019) [29]. The advantage of this procedure is the avoidance of a laparotomy. They describe improved intraoperative visibility, preservation of the integrity of the anterior rectus sheath, and reduced post-operative pain [29]. However, this procedure is newly developed, requiring more evidence to draw conclusions about the outcomes of a minimally invasive approach in comparison to the traditional open technique.

## 5. Conclusions

Despite a high risk of morbidity, pelvic exenteration remains the only possibility for cure in many patients with locally advanced or recurrent gynecologic malignancies. The use of myocutaneous flaps has been shown to improve patient outcomes and satisfaction. This case series confirms the feasibility of pelvic reconstruction with the use of RAM or RAMP flaps. Pelvic reconstruction at the time of radical resection should be considered in appropriate patients. The development of a team approach with input from both Gynecologic Oncology and Plastic Surgery is ideal, and consideration of other flaps may be better suited in appropriate patients.

## Figures and Tables

**Table 1:** Patient characteristics.

Patient Characteristics	*n* = 28
Age, median years (range)	57 (38–77)
Body Mass Index, median kg/m^2^ (range) ^1^	27 (22–50.9)
History of smoking, *n* (%)	
Yes	5 (17.9%)
No	17 (60.7%)
Unknown	6 (21.4%)
Cancer type, *n* (%)	
Vulvar	15 (53.6%)
Cervical	8 (28.6%)
Endometrial	2 (7.1%)
Vaginal	3 (10.7%)
Disease status, *n* (%)	
Primary	8 (28.6%)
Recurrent	20 (71.4%)
History of radiation treatment, *n* (%)	
Yes	24 (85.7%)
No	3 (10.7%)
Unknown	1 (3.6%)

^1^ *n* = 14.

**Table 2:** Surgical characteristics.

Surgical Characteristics	*n* = 28
Type of surgery, *n* (%)	
Total exenteration	8 (28.6%)
Anterior exenteration	4 (14.3%)
Posterior exenteration	8 (28.6%)
Radical vulvectomy	6 (21.4%)
Radical vaginectomy	2 (7.1%)
Type of myocutaneous flap, *n* (%)	
Vertical RAM	6 (21.4%)
Transverse RAM	18 (64.3%)
RAMP	4 (14.3%)
Concurrent procedures, *n* (%)	
Colostomy	11 (39.3%)
Urinary reconstruction	14 (50.0%)
Neovagina	14 (50.0%)
Rectal anastomosis with ileostomy	5 (17.9%)
None	5 (17.9%)
Estimated blood loss, median ml (range)	1000 (200–4500)
Intraoperative blood transfusion, *n* (%)	
Yes	8 (28.6%)
No	16 (57.1%)
Unknown	4 (14.3%)
Operative time, median min (range)	480 (240–780)
Surgical service for flap, *n* (%)	
Gynecologic Oncology	26 (92.9%)
Plastic Surgery	2 (7.1%)
Length of admission, median days (range)	16 (5–39)

Abbreviations: RAM, rectus abdominis myocutaneous flap; RAMP, rectus abdominis myoperitoneal flap; mL, milliliter; min, minutes.

**Table 3:** Flap-specific complications and outcomes.

Complications	*n* = 28
Recipient-site complications, *n* (%) ^1^	
Flap cellulitis	4 (14.3%)
Flap breakdown	5 (17.9%)
Flap necrosis	5 (17.9%)
Donor-site complications, *n* (%) ^1^	
Infection	7 (25.0%)
Necrosis	3 (10.7%)
Hernia	0 (0.0%)
Neovaginal complications, *n* (%) *	
Stenosis	2 (14.3%)
Rectovaginal fistula	3 (21.4%)
Recurrence, *n* (%)	
Yes	14 (50%)
No	14 (50%)
Location of the recurrence, *n* (%) *	
Perineal	5 (35.7%)
Pelvic	3 (21.4%)
Distant	6 (42.9%)
Status at last follow up, *n* (%)	
Alive without disease	14 (50%)
Alive with disease	8 (28.6%)
Death due to disease	3 (10.7%)
Death due to other causes	3 (10.7%)

^1^ More than one complication can occur in each patient. * *n* = 14.

## Data Availability

The data presented in this study are available on request from the corresponding author. The data are not publicly available due to privacy restrictions.
